# Systematic review of the current status of cadaveric simulation for surgical training

**DOI:** 10.1002/bjs.11325

**Published:** 2019-10-01

**Authors:** H. K. James, A. W. Chapman, G. T. R. Pattison, D. R. Griffin, J. D. Fisher

**Affiliations:** ^1^ Clinical Trials Unit, Warwick Medical School University Hospitals Coventry and Warwickshire Coventry UK; ^2^ Department of Trauma and Orthopaedic Surgery University Hospitals Coventry and Warwickshire Coventry UK

## Abstract

**Background:**

There is growing interest in and provision of cadaveric simulation courses for surgical trainees. This is being driven by the need to modernize and improve the efficiency of surgical training within the current challenging training climate. The objective of this systematic review is to describe and evaluate the evidence for cadaveric simulation in postgraduate surgical training.

**Methods:**

A PRISMA‐compliant systematic literature review of studies that prospectively evaluated a cadaveric simulation training intervention for surgical trainees was undertaken. All relevant databases and trial registries were searched to January 2019. Methodological rigour was assessed using the widely validated Medical Education Research Quality Index (MERSQI) tool.

**Results:**

A total of 51 studies were included, involving 2002 surgical trainees across 69 cadaveric training interventions. Of these, 22 assessed the impact of the cadaveric training intervention using only subjective measures, five measured impact by change in learner knowledge, and 23 used objective tools to assess change in learner behaviour after training. Only one study assessed patient outcome and demonstrated transfer of skill from the simulated environment to the workplace. Of the included studies, 67 per cent had weak methodology (MERSQI score less than 10·7).

**Conclusion:**

There is an abundance of relatively low‐quality evidence showing that cadaveric simulation induces short‐term skill acquisition as measured by objective means. There is currently a lack of evidence of skill retention, and of transfer of skills following training into the live operating theatre.

## Graphical abstract

This systematic review of cadaveric simulation for surgical training showed that there is low‐quality evidence of short‐term skill acquisition after training, as measured by objective means. There is a lack of evidence of skill retention longitudinally.

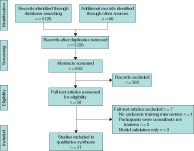

Making a comeback?

## Abstract (es)

**Antecedentes:**

Existe un interés creciente en los cursos de simulación con cadáveres para la formación de residentes en cirugía, así como en aumentar la disponibilidad de dichos cursos. Ello es debido a la necesidad de modernizar y mejorar la eficiencia del entrenamiento en el marco actual del reto que supone la formación quirúrgica. El objetivo de esta revisión sistemática era describir y evaluar la evidencia del uso de la simulación con cadáveres en la formación quirúrgica de posgrado.

**Métodos:**

Siguiendo la normativa PRISMA, se realizó una revisión sistemática de la literatura

de estudios que evaluaban prospectivamente el entrenamiento quirúrgico mediante la simulación con cadáveres. Se realizaron búsquedas en todas las bases de datos relevantes y en registros de ensayos clínicos hasta enero de 2019. Se evaluó el rigor metodológico utilizando la herramienta MERSQI (*Medical Education Research Quality Index*), ampliamente validada.

**Resultados:**

Se incluyeron 51 estudios, con un total de 2.002 residentes de cirugía y 69 intervenciones de formación con simulación con cadáveres. Del total de dichos estudios, 22 evaluaron el impacto de la cirugía con cadáver utilizando solo medidas subjetivas, 5 midieron el impacto por el cambio en el conocimiento del alumno y 23 utilizaron herramientas objetivas para evaluar el cambio en el comportamiento del alumno después de la formación. Solo un estudio evaluó el resultado en pacientes, demostrando la transferencia de habilidades del entorno simulado al lugar de trabajo. De los estudios incluidos, el 55% tenía una metodología débil (puntuación MERSQI < 10,7).

**Conclusión:**

Existe amplia evidencia, pero de baja calidad, referente a la simulación con cadáveres en la formación quirúrgica, demostrando que esta popular herramienta puede ser útil para adquirir habilidades a corto plazo, tal como indican los resultados derivados de medidas objetivas. Actualmente hay una falta de evidencia basada en estudios longitudinales respecto a la retención de habilidades y de cómo estas se transfieren al quirófano real una vez finalizada la formación. Se requieren futuros ensayos aleatorizados de alta calidad para abordar este punto.

## Introduction

There is growing interest in the use of cadaveric simulation in postgraduate surgical training^1^. The move to incorporate simulation into surgical training is driven by a need to improve training efficiency in the current climate of reduced working hours^2^, ^3^, financial constraint and emphasis on patient safety^4^. Cadaveric simulation is of particular interest, as it provides ultra‐high‐fidelity representation of surgical anatomy as encountered *in vivo* 1, 5, authentic tissue handling^6^ and complex three‐dimensional neurovascular relationships, which are difficult to appreciate in textbooks and almost impossible to replicate in synthetic models^7^. Cadaveric simulation offers the opportunity to practise an operation in its entirety with high environmental, equipment and psychological fidelity, thereby enabling the rapid acquisition of procedural skills^5^ and attainment of competence in a setting remote from patient care^8^. With the current increase in availability of cadaveric training courses for surgical trainees^9^, a systematic evaluation of the evidence for their use is both timely and necessary.

The purpose of this review was to describe and evaluate the evidence for the use of cadaveric simulation in postgraduate surgical training.

## Methods

This systematic review was conducted in accordance with the PRISMA guidelines^10^, ^11^; the review protocol was registered with PROSPERO (an international prospective register of systematic reviews)^12^.

### Search strategy and data sources

A literature search was conducted in January 2019 using MEDLINE (Ovid) (1946 to the present), CINAHL (EBSCO) (Cumulative Index of Nursing and Allied Health Literature), Centre for Reviews and Dissemination Database, ISRCTN Registry, Cochrane Central Register of Controlled Trials, NHS Evidence, PubMed (1950 to the present), Embase (Ovid) (1947 to the present), Scopus, Australian Clinical Trials Registry and Google Scholar. Medical Subject Headings (MeSH) terms and text words from the MEDLINE search strategy (*Table* *S1*, supporting information) were adapted for other databases according to the required syntax.

Search results were limited to human subjects and the English language. Duplicates were removed, and retrieved titles and abstracts were screened for initial eligibility. Reference lists of included studies and old reviews were hand‐searched to ensure literature saturation.

### Selection criteria and data extraction

The initial eligibility screening criteria were: study participants were postgraduate doctors in training; there was exposure to human cadaveric simulation training; and there was an attempt at measuring the educational impact.

Studies were excluded at screening if they used animal cadaveric models, involved veterinary trainees, or were purely descriptive feasibility studies describing a cadaveric technique, with no assessment of the educational impact.

Abstracts that passed eligibility screening were retrieved in full text. Reference lists of full‐text articles were examined for relevant studies, and those found by hand‐searching were subject to the same eligibility screening process.

The data were extracted from the full‐text articles using piloted data extraction forms, by two reviewers working independently. Data items collected included: participant characteristics (number, stage of training and specialty); study characteristics (single‐centre *versus* multicentre, eligibility criteria defined, loss to follow‐up); cadaveric training (intervention, cadaveric model used, skills taught, comparator group (where applicable)); assessment of educational impact (primary outcome measure, evidence of instrument validation, results summary (objective and subjective), post‐test assessment and evidence of skill transfer (if applicable)).

### Data analysis, quality assessment and evidence synthesis

Included studies were assigned a level of evidence score using a modified version of the Oxford Centre for Evidence‐Based Medicine (OCEBM) classification, which has been adapted by the European Association of Endoscopic Surgery and is used widely in educational systematic reviews^13^, ^14^. Methodological rigour of included studies was scored using the Medical Education Research Quality Instrument (MERSQI), which is a previously validated assessment tool^15^, ^16^, ^17^ for quantitative appraisal of medical education research across six domains: study design, sampling, type of data, instrument validity, data analysis and outcome. The maximum score is 18 points. The mean MERSQI score of both independent assessors for each included study is reported.

**Figure 1:**
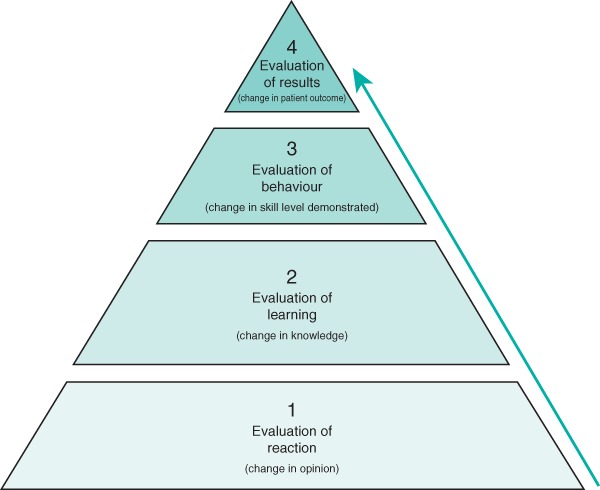
Adapted Kirkpatrick hierarchy for assessing educational impact

A qualitative, narrative synthesis of evidence was undertaken, structured around an adapted Kirkpatrick's hierarchy for assessing the educational impact of a teaching intervention (*Fig*. 1).

## Results

**Figure 2:**
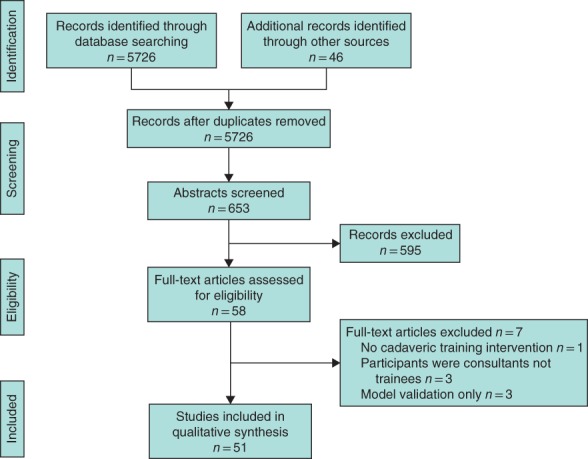
PRISMA diagram of included studies

The initial search generated 5726 results, of which 5073 were clearly ineligible and rejected at title review (*Fig*. 2). A total of 653 abstracts were screened, 595 of which did not pass eligibility screening and were excluded. Some 58 articles were accessed in full text and reviewed carefully; one study was rejected at this stage as there was no cadaveric simulation training intervention, three were rejected as the study participants were consultants not trainees, and three studies were rejected as there was cadaveric model validation only with no assessment of educational impact. Fifty‐one studies were included in the review, of which 47 were full‐text original research articles and four were conference posters. The main characteristics of studies, including OCEBM and mean MERSQI scores are shown in *Tables* *S2* *–* *S5* (supporting information)^18^, ^19^, ^20^, ^21^, ^22^, ^23^, ^24^, ^25^, ^26^, ^27^, ^28^, ^29^, ^30^, ^31^, ^32^, ^33^, ^34^, ^35^, ^36^, ^37^, ^38^, ^39^, ^40^, ^41^, ^42^, ^43^, ^44^, ^45^, ^46^, ^47^, ^48^, ^49^, ^50^, ^51^, ^52^, ^53^, ^54^, ^55^, ^56^, ^57^, ^58^, ^59^, ^60^, ^61^, ^62^, ^63^, ^64^, ^65^, ^66^, ^67^, ^68^.

### Study design and setting

Eight studies were RCTs, six were comparative cohort studies, and 37 were non‐comparative cohort studies.

The majority of studies were from the USA (35 studies) and the UK (8), with the remainder from Canada (4), Australia (2) and one each from Germany and Japan. All studies, except one^33^ were delivered at a single centre.

### Participants

The number of participants in the included studies ranged from three to 390, totalling 2002 individual participants across 69 cadaveric training interventions, representing the breadth of surgical training grades.

### Surgical specialty

In total, 12 surgical specialties and subspecialties were included (*Tables* *S2* *–* *S5*). Most studies were within general surgery (14), trauma and orthopaedic surgery (9) and neurosurgery (7). All studies were single‐speciality.

### Study quality

The mean MERSQI score was 9·4 (range 5–14). In terms of level of evidence, only two of 51 studies were OCEBM level 1b (RCT of good quality and adequate sample size with a power calculation), six studies were OCEBM 2a (RCT of reasonable quality and/or of inadequate sample size), six were OCEBM 2b (parallel cohort study), and 37 were OCEBM level 3 (non‐randomized, non‐comparative trials, descriptive research).

A linear relationship was observed between Kirkpatrick level and mean MERSQI score, suggesting that quality of evidence is linked with robust methodology.

### Measurement of educational impact

An assessment of educational impact of the training intervention was made using objective measures in 28 of the 51 included studies, and using subjective measures only in the other 23 studies. Sixteen of the 28 studies that used objective outcome measures attempted to measure skill transfer after training.

#### *Level 1: Reaction*

Twenty‐two of the 51 studies measured the educational impact of a cadaveric training intervention using subjective measures of learner reaction/opinion. One^18^ of these studies used a comparative cohort design, comparing cadaveric‐trained with virtual reality‐trained participants (OCEBM level 2b), and 21^19^, ^20^, ^21^, ^22^, ^23^, ^24^, ^25^, ^26^, ^27^, ^28^, ^29^, ^30^, ^31^, ^32^, ^33^, ^34^, ^35^, ^36^, ^37^, ^38^, ^39^ were descriptive research studies using non‐randomized, non‐comparative methods (OCEBM level 3). Two^19^, ^29^ of the Kirkpatrick level 1 studies attempted to measure skill transfer following the cadaveric training intervention. All 22 studies used participant questionnaires to assess learner reaction, most of which were purpose‐designed^38^ and not validated formally. The outcome measures included learner reaction with respect to simulation fidelity, learner opinion on the usefulness of the training, and change in operative confidence and self‐perceived competency after the training. All level 1 studies reported a positive effect of the cadaveric simulation training as measured by learner reaction/opinion.

#### *Level 2: Learning*

Five studies assessed the educational impact of the cadaveric training intervention by measuring change in learner knowledge. One^40^ of these studies was an RCT comparing cadaveric simulation training with a low‐fidelity bench‐top simulator, one^41^ was a cohort study comparing learning in cadaveric‐trained participants with those who received didactic teaching materials only, and three^42^, ^43^, ^44^ were non‐comparative cohort studies. Three studies^40^, ^43^, ^44^ used procedural knowledge scores as the primary outcome measure, and two^41^, ^42^ used *viva voce* and oral checklist examinations.

Cadaveric simulation training made no difference to the postintervention test scores in the study of AlJamal and colleagues^40^, but a significant improvement in overall examination scores was seen in the cadaveric‐trained group by Sharma and co‐workers^41^. Significant improvement in post‐test knowledge scores was reported in the three non‐comparative studies^42^, ^43^, ^44^ following cadaveric training.

#### *Level 3: Behaviour*

Twenty‐three studies assessed the educational impact of a cadaveric training intervention by attempting to measure a change in learner behaviour. Objective assessment methods of learner behaviour were highly variable, and included operational metrics (such as procedure time, error rate, hand motion analysis, path length) and final product analysis. Various score‐based methods were also used, including procedure scores, global rating scale (GRS), OSATS (Objective Structured Assessment of Technical Skills in Surgery) and the GOALS (Global Operative Assessment of Laparoscopic Skills) scale. Seven^45^, ^46^, ^47^, ^48^, ^49^, ^50^, ^51^ of the 23 studies were RCTs and 16^52^, ^53^, ^54^, ^55^, ^56^, ^57^, ^58^, ^59^, ^60^, ^61^, ^62^, ^63^, ^64^, ^65^, ^66^, ^67^ were cohort studies. Of the seven RCTs, three^45^, ^46^, ^47^ compared cadaveric simulation with no simulation training, and four^48^, ^49^, ^50^, ^51^ compared cadaveric simulation with low‐fidelity simulation.

Compared with no simulation training, cadaveric simulation‐trained learners showed significant improvement in most of the tested skill domains^45^, ^46^, ^47^. When comparing behaviour change after training in low‐fidelity simulation‐trained and cadaveric simulation‐trained learners, the results were mixed. Camp *et al*.^51^ reported that cadaveric training was superior to virtual reality (VR) when teaching knee arthroscopy, with greater improvement in procedural rating scores and reduced procedure time seen in the cadaveric‐trained compared with the VR‐trained group. Sidhu and colleagues^49^ reported that cadaveric training was superior to a bench‐top simulator for teaching graft‐to‐arterial anastomosis, as measured by a task‐based checklist (TBC), GRS and final product analysis (FPA). A greater benefit of the cadaveric training was seen in the more junior study participants.

Conversely, Anastakis and co‐workers^48^ compared behaviour change in cadaveric‐trained, low‐fidelity bench model‐trained and written materials only‐trained groups of learners performing basic general surgical skills, measured by procedural checklist scores and GRS. They found that the bench‐ and cadaveric‐trained groups performed better than the written materials only group, and that performances of the cadaveric‐ and bench‐trained groups were equivalent. Gottschalk *et al*.^50^ compared the performance of cadaveric‐trained, low‐fidelity bench model‐trained and ‘no training’ groups at cervical lateral mass screw placement using FPA. They found that, although both the cadaveric‐ and bench‐trained groups outperformed the no training group, the bench‐trained group had greater improvement in performance.

Of the 16 cohort studies measuring change in learner behaviour, five were comparative in design. Three studies^53^, ^54^, ^55^ compared inexperienced *versus* experienced performance, one^52^ compared behaviour change in cadaveric simulation‐trained *versus* low‐fidelity simulation‐trained cohorts, and one^56^ compared within‐subject performance change after cadaveric simulation training. Eleven^57^, ^58^, ^59^, ^60^, ^61^, ^62^, ^63^, ^64^, ^65^, ^66^, ^67^ were non‐comparative descriptive studies.

The primary objective of the three studies comparing inexperienced and experienced performance was construct validation of the simulator and/or assessment tools used in the studies. Zirkle and colleagues^53^ found that, when performing cortical mastoidectomy in a cadaveric simulation setting, FPA did not correlate with trainee experience, but GRS and TBC scores did. Mednick and co‐workers^55^ also found that, when performing corneal rust ring removal, FPA did not correlate with trainee experience, although procedure time did. Mackenzie *et al*.^54^ compared preintervention, immediately after (less than 4 weeks) and delayed (12–18 months) intervention scores for cadaveric‐trained, inexperienced learners with experienced ‘expert’ performance when undertaking lower‐extremity vascular exposure, repair and fasciotomy. The outcome measures were TBC, GRS, error frequency and procedure time. The results showed that experienced performance was significantly better at all time points, that performance amongst the inexperienced group was highly variable, and that evidence of skill retention was seen at 18 months postintervention.

When comparing cadaveric simulation‐trained and low‐fidelity (VR) simulation‐trained cohorts performing laparoscopic sigmoid colectomy, LeBlanc *et al*.^52^ reported that technical skills scores were better in the low‐fidelity group.

Of the 11 non‐comparative descriptive studies, all reported improvement in trainee performance after cadaveric simulation training, using a variety of outcome measures such as FPA, GRS and operational metrics.

#### *Level 4: Objective measurement of educational impact by change in patient outcome*

Only one^68^ of the 51 studies included in this review assessed the impact of the cadaveric training intervention on real‐world patient outcomes. Martin and colleagues^68^ measured the impact of cadaveric training on the real‐world performance of ‘core’ invasive skills (endotracheal tube insertion, chest tube insertion and venous cut‐down) by eight surgical trainees during the first 3 months of a trauma rotation. The complication rate for all skills decreased significantly immediately and at 3 weeks after instruction (*P* < 0·02). Initial trauma resuscitation time after training decreased from approximately 25 to 10 min in 80 patients treated by the participants^68^. The authors concluded that trainees' skills improve rapidly with competency‐based instruction (CBI), skills learnt through CBI in the laboratory can be translated to and sustained in the clinical setting, and CBI yields competent trainees who perform skills rapidly and with minimal complications.

### Cadaveric models used for simulation training

A wide variety of cadaveric models were used in the included studies. Three studies^26^, ^35^, ^67^ used innovative techniques to perfuse or reconstitute cadaveric material for training purposes, to improve the fidelity of the simulation. These studies were all in the field of neurosurgery, and involved cannulation of the great vessels of the neck to allow pulsatile perfusion of cadaveric heads with an artificial blood substitute. All reported very high learner satisfaction with the models, and recognition of the opportunity that live reconstitution offers for overcoming the criticism^35^, ^67^ of conventional, non‐perfused cadaveric material, in that it does not bleed and thus the simulation fidelity is limited for teaching procedures where bleeding is a potential major consequence.

Fresh cadavers were used in 12^20^, ^23^, ^28^, ^33^, ^37^, ^43^, ^48^, ^52^, ^54^, ^55^, ^65^, ^68^ of the reviewed studies. These offer the most authentic tissue‐handling fidelity^69^, but have the significant disadvantage of rapid deterioration, and therefore a short time‐window for their potential use. Use of fresh cadavers for simulation training relies on a regular, local system of body donation bequests, as they are typically used within 48 h of the donor's death, and certainly no more than 7 days later, which places logistical and infrastructure challenges on training providers.

Fresh‐frozen cadavers, used in 12^18^, ^21^, ^27^, ^30^, ^31^, ^32^, ^39^, ^42^, ^45^, ^46^, ^56^, ^57^ of the studies, have gained popularity due to their versatility. The cadavers are non‐exsanguinated, washed with antiseptic soap, and frozen to −20°C within 1 week of procurement^70^. Typically, around 3 days before use they are gradually thawed at room temperature, retaining the realistic tissue‐handling characteristics that are important for high‐fidelity simulation. Fresh‐frozen cadavers have the great advantage of being able to be refrozen and thawed at a later date, permitting reuse across multiple training interventions and thus maximizing potential use and cost‐efficiency^70^.

Soft‐fix Thiel embalming techniques were used in two studies^34^, ^58^. This technique seeks a method of cadaveric preservation that preserves tissue‐handling, enables longevity of specimen use, and avoids the occupational and environmental health risks associated with exposure to formaldehyde^71^. Organs and tissues retain their flexibility, and the colour of the tissue remains similar to that seen *in vivo*.

Only one study^41^ used traditional formalin‐fixed cadavers. Formalin has the advantages of being relatively inexpensive and widely available, with a long history of use in preserving cadavers for the purposes of teaching anatomy^71^. It does, however, lead to changes in the colour, strength and tissue‐handling characteristics of the cadaveric material^72^, which may limit its usefulness in surgical training. The study by Sharma *et al*.^41^ did not evaluate the fidelity of the simulation or discuss the rationale or impact on the educational value of the training as a result of using formalin‐fixed material. Twenty studies provided no information on the type of cadaveric material used in the training intervention.

Almost half of the included studies (22 of 51, 43 per cent) assessed the impact of the cadaveric training using subjective measures only, representing the lowest level of impact in educational research (Kirkpatrick level 1). The second most prevalent category was studies measuring the impact of the training intervention by learner behaviour change (23 of 51, 45 per cent) (Kirkpatrick level 3). Of these, seven were RCTs and 16 were cohort studies. Of the 23 studies assessing behaviour change following cadaveric training, only one^65^ measured behaviour change in the workplace; the rest measured behaviour change in the simulation laboratory. Only one^68^ of the 51 studies actually measured a change in patient outcome as a result of the cadaveric training intervention; this is the highest level of impact assessment in educational research (Kirkpatrick level 4).

## Discussion

The objective of this systematic review was to describe and evaluate evidence for the current use of cadaveric simulation in postgraduate surgical training. Fifty‐one studies involving 2002 surgical trainees across 69 cadaveric training interventions were included. Although there was research activity encompassing the breadth of surgical specialties, most studies were within general surgery (14), trauma and orthopaedic surgery (9) and neurosurgery (7). The majority were conducted in the USA (35) and UK (8). A wide range of methodology was used. Eight of 51 studies were RCTs (OCEBM level 1b and 2a), six were parallel cohort studies (OCEBM level 2b) and 37 were non‐comparative descriptive studies (OCEBM level 3).

There is evidence from three RCTs^45^, ^46^, ^47^ that cadaveric simulation training is superior to no training, yet the important question remains whether it is superior to low‐fidelity simulation training. This is of interest because cadaveric simulation training is expensive, and therefore needs to have demonstrable superiority over less expensive alternatives. The four RCTs^48^, ^49^, ^50^, ^51^ in this area revealed a mixed picture: two^49^, ^51^ showed superiority of cadaveric simulation training, one^48^ showed equivalence with low‐fidelity bench‐model training, and one^50^ showed that cadaveric simulation training was inferior to the bench‐model alternative.

The mean overall MERSQI score correlated well with the Kirkpatrick level: the higher the level of impact measured, the better the study methodology. Previous predictive studies^17^ have shown that studies with a MERSQI score of 10·7 or above are indicative of a methodologically strong study, likely to be accepted for publication. Some 67 per cent of the studies in this review had a MERSQI score below 10·7, and therefore the majority of the included studies could be considered to have weak methodology. Methodological problems noted amongst the included studies were: predominance of single‐site studies, lack of randomization, lack of comparator group, small sample sizes and underpowering, inadequate or absent reporting of descriptive statistics, overuse of a single group, and predominance of pretest/post‐test assessment strategies to determine the impact of the training intervention, which can overestimate the observed effect size^73^.

Several studies reported mixed‐modality training interventions, making assessment of the cadaveric component of the training in isolation impossible. There was also inadequate or absent description of the cadaveric model used in more than one‐third of the studies, which renders results pertaining to the face and content validity of training impossible to assess.

Half of the studies were published in the last 3 years, reflecting the recent explosion in popularity of cadaveric simulation training. Despite the clear attraction of cadaveric simulation training as measured by subjective means, there remains a dearth of evidence that there is retention and translation of skills learnt in the cadaveric laboratory into the operating theatre. The major ongoing challenge within educational research is demonstrating effective, sustained changes in learner behaviour, and improved patient outcome following a training intervention^73^. These challenges are particularly acute in the field of cadaveric simulation because of the cost and infrastructure demands, and there is presently little evidence that surgical educators are rising to meet this challenge.

This review has shown that there is an abundance of relatively low‐quality evidence indicating that cadaveric simulation may induce short‐term skill acquisition as measured by objective means. Adequately powered studies are needed to show whether skills are retained and transferable into the operating theatre before major investment in cadaveric simulation for surgical training can be recommended.

## Disclosure

The authors declare no conflict of interest.

## Supporting information


**Table S1** MEDLINE search strategy
**Table S2** Kirkpatrick Level 1: Studies that subjectively measure the impact of the training intervention by learner opinion
**Table S3** Kirkpatrick Level 2: Studies that objectively measure the impact of the training intervention by learner knowledge
**Table S4** Kirkpatrick Level 3: Studies that objectively measure the impact of the training intervention by change in learner behaviour
**Table S5** Kirkpatrick Level 4: Studies that objectively measure the impact of the training intervention by change in patient outcome
