# Supplementary material for: Systematic review of the current status of cadaveric simulation for surgical training
Source: Br J Surg. 2019 Oct 1;106(13):1726–34. doi: 10.1002/bjs.11325 (PMC6900127; doi:10.1002/bjs.11325)
Supplement: Supplementary file 1 — Table S1 MEDLINE search strategy Table S2 Kirkpatrick Level 1: Studies that subjectively measure the impact of the training intervention by learner opinion Table S3 Kirkpatrick Level 2: Studies that objectively measure the impact of the training intervention by learner knowledge Table S4 Kirkpatrick Level 3: Studies that objectively measure the impact of the training intervention by change in learner behaviour Table S5 Kirkpatrick Level 4: Studies that objectively measure the impact of the training intervention by change in patient outcome [file BJS-106-1726-s001.docx]

**BJS_11325**

**Systematic review of the current status of cadaveric simulation for surgical training**

**H. K. James, A. W. Chapman, G. T. R. Pattison, D. R. Griffin and J. Fisher**

| **Table S1 MEDLINE search strategy** |
| --- |
| **Category A**: ‘cadaver’ OR ‘cadaver*.mp’  **Category B**: ‘Clinical Competence/ OR Simulation Training/OR simulat*.mp  **Category C**: ‘Internship and Residency’/OR Clinical Competence/ OR Curriculum/ or surgical training.mp. OR Education, Medical, Graduate/. |
| Limits: Human Subjects and English Language. No date limits applied |

Categories A, B and C were combined with the Boolean operator AND and the specified limits applied

| **Table S2 Kirkpatrick Level 1: Studies that subjectively measure the impact of the training intervention by learner opinion** | | | | | | | |
| --- | --- | --- | --- | --- | --- | --- | --- |
| Study | Training Intervention | Skills Taught | Comparator | Primary Outcome Measure | Results | Skill Transfer | MERSQI Score |
| **OCEBM Level 2b: Comparative cohort design** | | | | | | | |
| *Sharma et a(18) 2012* General Surgery N=30 PGY 1-5 | Supervised performance of tasks on FFC | Level specific; senior = lap sigmoid colectomy, intermediate = lap incisional hernia repair, junior = basic laparoscopic tasks | VR trainer | Fidelity | CST perceived as significantly better model overall vs high fidelity VRS by all grades | N | 6 |
| **OCEBM Level 3: Non-randomised, non-comparative descriptive studies** | | | | | | | |
| *Dunnington et al(19), 2003*  General Surgery  N=9 PGY 2-3 | 2 hour skill session | Sentinel node mapping and excision, level I/level II axillary dissection | n/a | Fidelity, Operative Confidence, Learner Opinion | Valuable demonstration of procedure, practice opportunity and confidence | Y | 6 |
| *Gunst et al(20), 2009*, Trauma Surgery  N=18 PGY NS | 8 x 1 day course | Trauma surgical exposures | n/a | Operative confidence | Increase in confidence scores for 44/48 exposures (p<0.0001), no decline at 6/12 | N | 7.5 |
| *Reed et al(21), 2009*  Vascular Surgery  N = 45 PGY 2-3 | 6 discrete sessions over 24 months | Carotid endarterectomy, fem-pop bypass, SMA embolectomy, 4 compartment fasciotomy | n/a | Learner Opinion | 100% optimal training material, 97.8% rated the educational value of the course as perfect | N | 6 |
| *Lewis et al(22), 2012*  General Surgery  N = 150 PGY 1-5 | Weekly educational programme | Not specified | n/a | Leaner Opinion | Positive opinion of cadaveric sessions, learning stages of operations and increased confidence | N | 6 |
| *Sheckter et al(23), 2013*  Plastic Surgery  N = 192 PGY 1-6 | Weekly afternoon cadaveric lab | Curriculum based | n/a | Operative confidence | Overall confidence was improved, 1.90 to 4.20 (p<0.001) | N | 7 |
| *Gasco et al(24), 2013*  Neurosurgery  N=6 PGY NS | 57 cadaveric dissections within multi-modality simulation curriculum | Curriculum based | n/a | Learner Opinion | CST highest benefit vs other modalities (p<0.001). Effect was greater with junior vs senior | N | 10 |
| *Jansen et al(25), 2014*  Vascular Surgery  N = 26 PGY NS | 2 day cadaveric lab | Open vascular exposures | n/a | Leaner Opinion | 100% agreement with course meeting learner objectives | N | 6 |
| *Pham et al(26), 2014*  Neurosurgery  N = NS PGY 3-5 | Single procedural attempt on cadaver | Management of intraoperative ICA injury | n/a | Operative confidence and procedural knowledge | Improved knowledge and procedural confidence | N | 6.5 |
| *Ahmed et al(27), 2015*  Urology  N = 81 PGY 3-10 | 3 day modular cadaveric course | Curriculum based | n/a | Fidelity, Operative confidence, Learner opinion | All procedures scored a mean of 3/5 for face validity. Subjective improvement in skills and transferrable skills for the operating room | N | 6 |
| *Aydin et al(28), 2015*  Urology  N = 42 PGY NS | Urology Human Cadaver Training Programme | Not specified | n/a | Fidelity, Operative Confidence, Learner Opinion | Mean = 3/5 for face validity. Useful for learning anatomy, confidence boosted, skills improved and feasible for training | N | 6 |
| *Liu et al(29), 2015*  Neurosurgery  N = 12 PGY 2-7 | 4 or 5 x 4 hour sessions | Cranial and endonasal approaches, temporal drilling technique | n/a | Learner Opinion | Improved anatomy and liked rehearsal opportunity. 67% applied learning to real life operating | Y | 5 |
| *Clark et al(30), 2016*  Dermatologic surgery  N = 8 PGY 2-4 | Cadaveric lab practicum | Digital blocks, nail plate avulsions and nail matrix biopsies | n/a | Operative Confidence and Procedural Competency | Improvement in confidence and competency (post vs. pre) (p<0.0001 for both) | N | 7 |
| *Mahon et al(31), 2016*  Maxillofacial surgery  N = 41 PGY 3-7 | Cadaver course | Not stated | n/a | Operative Confidence | Senior participants confidence improved by 3 points, junior by 4 points | N | 6.5 |
| *Calio et al(32), 2017*  Neurosurgery  N = 17 PGY 1-5 | Not specified | Spinal decompression and fusions | n/a | Learner Opinion | Cadaveric training superior to other simulation modalities (94%) | N | 6 |
| *Lorenz et al(33), 2017*  General Surgery  N = 390 PGY NS | 0.5 day cadaveric lab within 3 day course | Open and endoscopic hernia repair | n/a | Learner Opinion | 95% stated improved training | N | 6 |
| *Nobuoka et al(34), 2017*  Hepatobiliary Surgery  N = NS PGY NS | Thiel cadaver workshop | Open + laparoscopic Whipple’s and major hepatectomy | n/a | Learner’s opinion | ‘most showed excellent satisfaction and good understanding of surgical anatomy was achieved in many participants | N | 3.5 |
| *Pacca et al(35), 2017*  Neurosurgery  N = NS PGY NS | ‘hands-on’ dissection course | Management of sharp and blunt ICA injury | n/a | Learner Opinions | 100% reported gaining new knowledge, learning new techniques and interest in further training on the model | N | 6 |
| *Takayesu et al(36), 2017*  Emergency Medicine  N = 22 PGY 3-4 | 1 hour cad session after mannequin teaching | Surgical cricothyroidotomy and tube thoracostomy | Low fidelity simulator and animal chest walls | Simulation fidelity and operative confidence | Higher fidelity of cadaveric training vs mannequins (p<0.0001) for both procedures | N | 7 |
| *Weber et al(37), 2017*  Plastic Surgery  N = 9 PGY 1-7 | Single procedural attempt within 7/7 of live attempt | Not stated | n/a | Operative Confidence | Improved median confidence score (pre-simulation 2/5 and post simulation 4/5) p<0.01 | N | 7 |
| *Bertolo et al(38), 2018*  Urology  N = 22 PGY 4-7 | 1 day intensive robotic course | Pelvic and kidney robotic procedures via the transperitoneal approach | n/a | Operative Confidence, Learner Opinion | Improved confidence in 5 robotic skill domains | N | 6.5 |
| *Chouari et al(39), 2018*  Plastic Surgery  N = 50 PGY 1-7 | Annual 2 day course, first day of 2 courses evaluated | Microsurgical flap reconstruction | n/a | Operative Confidence | Improved confidence (p<0.005) and preparedness for unsupervised bench work | N | 7.5 |

ASSET = Arthroscopic Surgical Skill Evaluation Tool EO = Expert Opinion GRS = Global Rating Scale

CST = Cadaveric Simulation Training ET = Endotracheal Tube Insertion OSATS = Objective structured Assessment of Technical Skill

CTB = Cadaveric Temporal Bone FFC = Fresh Frozen Cadaver TBC = Task Based Checklist

CT = Chest Tube Insertion FPA = Final Product Analysis VC = Venous Cutdown

| **Table S3 Kirkpatrick Level 2: Studies that objectively measure the impact of the training intervention by learner knowledge** | | | | | | | |
| --- | --- | --- | --- | --- | --- | --- | --- |
| Study | Training Intervention | Skills Taught | Comparator | Primary Outcome Measure | Results | Skill Transfer | MERSQI Score |
| **OCEBM Level 2a: Randomised Controlled Trial** | | | | | | | |
| *Al Jamal et al(40), 2018*  General Surgery  N = 14 PGY 1-4 | Single supervised performance on a cadaver | Endoscopic total inguinal hernia repair | Low fidelity bench top simulator | Procedural knowledge scores | No difference seen in test scores between two modalities | N | 10.5 |
| **OCEBM Level 2b: Comparative cohort studies** | | | | | | | |
| *Sharma et al(41), 2016*  General Surgery  N = 14 PGY 2-3 | 8 x 2 hour cadaveric lab sessions across 8 weeks | Open cervical, thoracic, abdominopelvic and extremity procedures | Course materials only | Viva Voce examination scores | Larger improvement in overall examination scores in cadaveric group (31% +/- 4% vs. 8%+/-3%, p=0.0006) | N | 9.5 |
| **OCEBM Level 3: Non-randomised, non-comparative descriptive studies** | | | | | | | |
| *Mitchell et al(42), 2012*  Vascular Surgery  N = 22 PGY 3-4 | 5 X 4 hour cadaveric lab sessions | Complex open vascular surgical approaches | n/a | Oral Checklist Exam | Mean examination scores significantly improved across all 5 exposures | N | 12 |
| *Robinson et al(43), 2017*  Vascular Surgery  N = 58 PGY 1-7 | 0.5 days cad lab as part of course | Open surgical approaches | n/a | Procedural knowledge scores | Improvement in knowledge scores in both groups | N | 7.5 |
| *Hazan et al(44), 2018*  Dermatologic Surgery  N = 40 PGY 1-4 | Cadaver training session | Not stated | n/a | Procedural knowledge scores | Overall improvement in pre and post intervention knowledge (p=0.001) | N | 8 |

ASSET = Arthroscopic Surgical Skill Evaluation Tool EO = Expert Opinion GRS = Global Rating Scale

CST = Cadaveric Simulation Training ET = Endotracheal Tube Insertion OSATS = Objective structured Assessment of Technical Skill

CTB = Cadaveric Temporal Bone FFC = Fresh Frozen Cadaver TBC = Task Based Checklist

CT = Chest Tube Insertion FPA = Final Product Analysis VC = Venous Cutdown

| **Table S4 Kirkpatrick Level 3: Studies that objectively measure the impact of the training intervention by change in learner behaviour** | | | | | | | |
| --- | --- | --- | --- | --- | --- | --- | --- |
| Study | Training Intervention | Skills Taught | Comparator | Primary Outcome Measure | Results | Skill Transfer | MERSQI Score |
| **Randomised Controlled Trials; Cadaveric Simulation vs. No Simulation** | | | | | | | |
| *Sharma et al(45), 2013*  General Surgery  N = 19 PGY 1-2 | 10 repetitions of 5 tasks across 2 days | Peg transfer, simulated appendicectomy, intra and extra corporeal knot tying | No training | GOALS scale performance on cadavers, pre-/post intervention VR performance | 4/5 tasks on cadavers showed significant improvement on learning curve analysis. Post-test VR assessment showed safety of cautery (p=0.4) and left arm path (p=0.047) to be significantly improved in the intervention group | Y | 14 |
| *Sundar et al(46), 2016*  Neurosurgery  N = 8 PGY 1-4 and 2 medical students | Cadaveric training course | Pedicle and lateral mass screw placement | Didactic teaching | Final Product Analysis, surgical error | Reduced surgical error in the cadaveric vs. control group (p=0.04). Screw placement was more optimal in cadaveric vs control group in cervical, thoracic and lumbar regions (p=0.02, 0.04 and 0.04) | N | 14 |
| *Chong et al(47), 2017*  O&G  N = 34 PGY 1-4 | 0.5 DAY cadaveric lab | Transobturator tape insertion | Didactic teaching | Procedure scores on low fidelity mannequin | Procedure scores improved in intervention group vs. controls p<0.01 | Y | 11 |
| **Randomised Controlled Trials; Cadaveric Simulation vs. Low-fidelity Simulation** | | | | | | | |
| *Anastakis et al(48), 2003*  General Surgery  N = 23 PGY 1 | 4 hour cadaveric lab session | Basic general surgical skills | Low fidelity simulator and text materials only | Procedure checklist score and GRS | Significant effect of training modality on checklist and GRS scores. Bench and cadaveric training were superior to text, and bench and cadaveric training were equivalent | Y | 14 |
| *Sidhu et al(49), 2007*  Vascular Surgery  N = 27 PGY 1-4+ | 3 hour cadaveric lab session | Graft-to-arterial anastomosis | Benchtop simulator | Procedural checklist, GRS, final product analysis | Juniors practicing on cadaveric model performed better on checklist (p=0.05) and final product analysis (p=0.04). Seniors practicing on cadaveric models scored better on final product analysis | Y | 14 |
| *Gottschalk et al(50), 2015*  T&O Surgery  N = 15 PGY 1-6 | Cadaveric workshop | Cervical lateral mass screw placement | 1. Sawbones workshop  2. No training | Final Product Analysis | Both sawbones and cadaveric trained groups improved vs. no training (p<0.0001), sawbone group had modestly higher improvement post intervention than cadaveric group (mean aggregate difference from perfect screw placement -8.2 degress and -7.2 degrees) | N | 14 |
| *Camp et al(51), 2016*  T&O surgery  N = 45 PGY 1-5 | 4 hour cadaveric lab | Knee arthoscopy | VR simulator and no training | Procedure time and checklist scores | Cadaveric trained group improved ASSET scores by 1.1 points per hour of training vs. 0.5 for VR group. Significant decrease in operating time seen in cadaveric group pre- vs post training (p=0.002) | Y | 14 |
| **OCEBM 2b: Parallel Cohort Studies; Cadaveric vs. Low-fidelity Simulation** | | | | | | | |
| *LeBlanc et al(52), 2010*  General Surgery  N = 28 PGY NS | Single performance on cadaver as part of 1 day course | Laparoscopic sigmoid colectomy | VR | OSATS assessment during training | Technical skills scores overall better on VR simulator than cadaver | N | 13 |
| **OCEBM 2b: Parallel Cohort Studies; Inexperienced vs. Experienced performance** | | | | | | | |
| *Zirkle et al(53), 2007*  ENT  N = 19 PGY 1-4 | Performance under exam conditions | Cortical Mastoidectomy | Experienced performance | GRS, Final Product Analysis, Task Based Checklist | GRS, TBC and EO correlated with trainee experience. FPA did not. TBC correlated with EO | N | 13 |
| *Mackenzie et al(54), 2017*  Trauma Surgery  N = 40 PGY3-6 | ASSET Course | Lower extremity vascular exposure, repair and fasciotomy | Pre, post (immediate <4/52), post (delayed 12-18mths) vs experts | Procedure scores, GRS, error, frequency, procedure time | Decreased errors from 60 to 19% after training, improved knowledge and procedural steps (p<0.001). Interval experience rather than time since training affected skill retention up to 18 months later | Y | 14 |
| *Mednick et al(55), 2017*  Ophthalmology  N = 11 PGY 2 | Single procedural attempt | Corneal rust ring removal | Experienced performance | Time error rate, final product analysis | Procedure time longer for inexperienced group (187 vs 117 secs mean), rust removal percentage similar (61 vs 69%) NSS No perforations | N | 10 |
| **OCEBM 2b: Parallel Cohort Studies; Within subject performance comparison** | | | | | | | |
| *Wong et al(56), 2004*  General Surgery  N = 9 PGY 2-4 | Cadaveric lab practicum with embedded study | Saphenous venous cutdown | n/a | Procedure time, final product analysis | Decreased mean incision size 4.6 vs 3.4cm (p<0.05), mean time taken to completion of procedure 360 vs 232 seconds (p<0.05), % subjects experiencing complications 37.5% vs 0% (<0.05) | N | 9.5 |
| **OCEBM 3: Non-comparative studies, descriptive research** | | | | | | | |
| *Rowland et al(57), 1994*  T&O Surgery  N = 12 PGY NS | Supervised task performance | Endoscopic carpal tunnel release | n/a | Final Product Analysis | 38% incomplete release of transverse carpal ligament, 17% showed complications | N | 8 |
| *Levine et al(58), 2006*  O&G  N = 29 PGY 2-3 | 5 half day cad sessions | Gynae laparoscopy, salpingectomy, salpingostomy | n/a | Operational metrics | Decrease in mean bead transfer time of 38.5 seconds (p=0.02), median increase in number of beads transferred 2.5 (p=0.0001), median decrease in suture time was 63.5 seconds (p=0.001) | Y | 9.5 |
| *Bergeson et al(59), 2008*  T&O surgery  N = 3 PGY 1+3 | Serial performance on 5 spines | Thoracic pedicle screw placement | n/a | Final product analysis | Decrease in the proportion of total screw violations by the third cadaver (p<0.001) and in critical screw violations by the fourth cadaver (p=0.01) | Y | 9.5 |
| *Tortolani et al(60), 2013*  T&O Surgery  N = 3 PGY NS | 2 cadaveric lab sessions | Thoracic pedicle screw placement | n/a | Final Product Analysis | Placement accuracy improved following training and structured feedback (44% accurate placement rate pre- and 58% post p=0.01) | N | 9.5 |
| *Mowry et al(61), 2014*  ENT  N = 56 PGY 1-4 | Weekly course over 9 months with self-directed access to cadaveric lab | Microdissection of temporal bone | n/a | PBA, GRS and performance time | Test scores from most recent resident year correlated well with number of CTB’s drilled during corresponding year (r=0.42, p=0.002) and strong correlation between score during the highest year of training and cumulative number of CTB’s drilled during residency (r=0.604, p=0.005) | N | 12.5 |
| *Awad et al(62), 2015*  ENT  N = 32 PGY 2-6 | 2 x 3 hour sessions | Mastoidectomy | n/a | Task specific | Longitudinal assessment showed significant improvement with iteration | Y | 12.5 |
| *Egle et al(63), 2015*  General Surgery  N = 14 PGY 1-5 | Cadaveric lab based anastomosis workshop, then repeated 1 week later | Vascular and hand-sewn bowel anastomosis | n/a | OSATS, procedure time and final product analysis | Vascular anastomosis, operating time decreased (NS), OSATS scores improved (NS) and final product analysis (anastomosis leak pressure 38.9 vs 71.8psi p=0.001) for bowel operating time decreased (23 vs 18 minutes p<0.001), OSATS scores improved (12.9 vs 14.4 p<0.001), leak pressures improved (17.7 vs 26.9psi p<0.001) | Y | 12.5 |
| *Nicandri et al(64), 2015*  T&O Surgery  N = 46 PGY NS | 3 Day AAOS fundamentals of arthroscopy course | Knee Arthroscopy | n/a | Operational metrics, Checklist scores | 9.2% improvement in mean ASSET score (p=0.001), biggest gain seen in those with less than 20 arthroscopic cases (13.2% improvement). Improvement seen in operational metrics post-training; time (18% decrease, p=0.1), path distance (13% decrease, p=0.2) probe path distance (16% decrease, p=0.05), smoothness (10% improvement, p=0.001), excessive probe pressure (improved 440%, p=0.02) | Y | 12.5 |
| *Kim(65), 2016*  General Surgery  N = 133 PGY 1-5 | 4-6 Cadaveric lab sessions with practice opportunities | General surgical core procedures, stratified by stage of training | n/a | GRS, modified OSATS | % skills that could be performed independently increased from 40-60% (p<0.04) | Y | 9.5 |
| *Martin et al(66), 2016*  T&O Surgery  N = 48 PGY 1-5 | 4 day course with didactics, low fidelity simulation and cadaveric lab | Knee and shoulder arthroscopy | n/a | Operational metrics using VR simulator pre-and post-test | Improvement post-training in all domains; tip probe distance, time to completion (p<0.001) | Y | 12 |
| *Ciporen et al(67), 2017*  Neurosurgery  N = 10, PGY NS | 3 attempts at procedure under supervision | Endoscopic management of iatrogenic carotid artery injury | n/a | Operational metrics, final product analysis | Time to control bleeding improved with repeated exposure. Senior residents performed better across all performance score domains | N | 8 |

ASSET = Arthroscopic Surgical Skill Evaluation Tool EO = Expert Opinion GRS = Global Rating Scale

CST = Cadaveric Simulation Training ET = Endotracheal Tube Insertion OSATS = Objective structured Assessment of Technical Skill

CTB = Cadaveric Temporal Bone FFC = Fresh Frozen Cadaver TBC = Task Based Checklist

CT = Chest Tube Insertion FPA = Final Product Analysis VC = Venous Cutdown

| **Table S5 Kirkpatrick Level 4: Studies that objectively measure the impact of the training intervention by change in patient outcome** | | | | | | | |
| --- | --- | --- | --- | --- | --- | --- | --- |
| Study | Training Intervention | Skills Taught | Comparator | Primary Outcome Measure | Results | Skill Transfer | MERSQI Score |
| *Martin et al(68), 1998*  General Surgery  N = 8 PGY 1 | Cadaveric lab session | Chest tube insertion, endotracheal tube insertion, venous cutdown | n/a | Correct performance of skill to completion without complication in 2 minutes | all residents passed the 3 skills immediately after instruction. At 3 weeks 8/8 passed VC and CT, 6/8 passed ET. Changes in performance over time for both intervals were statistically significant. Times improved after immediate instruction and at 3 weeks (except 2 residents (one each in CT and VC) and at more than 3 weeks (except one resident in VC) Change in time for both intervals was statistically significant (p<0.05). Complications for all skills decreased significantly immediately and at 3 weeks after instruction (p<0.02) | Y | 13.5 |

ASSET = Arthroscopic Surgical Skill Evaluation Tool EO = Expert Opinion GRS = Global Rating Scale

CST = Cadaveric Simulation Training ET = Endotracheal Tube Insertion OSATS = Objective structured Assessment of Technical Skill

CTB = Cadaveric Temporal Bone FFC = Fresh Frozen Cadaver TBC = Task Based Checklist

CT = Chest Tube Insertion FPA = Final Product Analysis VC = Venous Cutdown
